# Microstate Analysis Reflects Maturation of the Preterm Brain

**DOI:** 10.1007/s10548-023-01008-0

**Published:** 2023-10-12

**Authors:** Tim Hermans, Mohammad Khazaei, Khadijeh Raeisi, Pierpaolo Croce, Gabriella Tamburro, Anneleen Dereymaeker, Maarten De Vos, Filippo Zappasodi, Silvia Comani

**Affiliations:** 1 Department of Electrical Engineering (ESAT), STADIUS Center for Dynamical Systems, Signal Processing and Data Analytics, KU Leuven, Leuven, Belgium; 2 Department of Neuroscience Imaging and Clinical Sciences, G. d’Annunzio University of Chieti–Pescara, Chieti, Italy; 3 Behavioral Imaging and Neural Dynamics Center, G. d’Annunzio University of Chieti–Pescara, Chieti, Italy; 4 Department of Development and Regeneration, KU Leuven, Leuven, Belgium; 5 Neonatal Intensive Care Unit, UZ Leuven, Leuven, Belgium; 6 Institute for Advanced Biomedical Technologies, G. d’Annunzio University of Chieti–Pescara, Chieti, Italy

**Keywords:** Neonatal EEG, Microstate analysis, Brain maturation, Quantitative EEG, Biomedical signal processing

## Abstract

Preterm neonates are at risk of long-term neurodevelopmental impairments due to disruption of natural brain development. Electroencephalography (EEG) analysis can provide insights into brain development of preterm neonates. This study aims to explore the use of microstate (MS) analysis to evaluate global brain dynamics changes during maturation in preterm neonates with normal neurodevelopmental outcome.

The dataset included 135 EEGs obtained from 48 neonates at varying postmenstrual ages (26.4 to 47.7 weeks), divided into four age groups. For each recording we extracted a 5-minute epoch during quiet sleep (QS) and during non-quiet sleep (NQS), resulting in eight groups (4 age group x 2 sleep states). We compared MS maps and corresponding (map-specific) MS metrics across groups using group-level maps. Additionally, we investigated individual map metrics.

Four group-level MS maps accounted for approximately 70% of the global variance and showed non-random syntax. MS topographies and transitions changed significantly when neonates reached 37 weeks. For both sleep states and all MS maps, MS duration decreased and occurrence increased with age. The same relationships were found using individual maps, showing strong correlations (Pearson coefficients up to 0.74) between individual map metrics and post-menstrual age. Moreover, the Hurst exponent of the individual MS sequence decreased with age.

The observed changes in MS metrics with age might reflect the development of the preterm brain, which is characterized by formation of neural networks. Therefore, MS analysis is a promising tool for monitoring preterm neonatal brain maturation, while our study can serve as a valuable reference for investigating EEGs of neonates with abnormal neurodevelopmental outcomes.

**Supplementary Information:**

The online version contains supplementary material available at 10.1007/s10548-023-01008-0.

## Introduction

The human brain undergoes rapid maturation and development during the second half of gestation (i.e., from 20 to 40 weeks of gestation). The physiological changes that occur in this period include the brain growth, realized through myelination and glial cell proliferation and differentiation, and the formation of functional neural networks, achieved through synaptogenesis and synaptic pruning (Back and Miller 2014; Kinney and Volpe 2012). Preterm birth disrupts this natural progression of brain development and puts the infant at risk of developing long-term neurodevelopmental impairments (Back 2017; Volpe 2009, 2019).

Analysing the brain dynamics of preterm neonates provides valuable insights in the maturation of the neonatal brain when the neonate is under surveillance in the neonatal intensive care unit (NICU). The most common method used to monitor the functional changes in the preterm neonatal brain is electroencephalography (EEG), which non-invasively measures the electrical activity of the brain. Previous studies have shown that EEG can detect abnormalities in the functional connectivity and activity of the neonatal brain and can be used to track brain maturation. Those studies focused on analysing the background EEG (i.e., not task-related electrical activity of the brain) in terms of continuity, frequency content, coherence, connectivity and complexity (De Wel et al. 2017; Dereymaeker et al. 2016, 2017a; Lavanga et al. 2017, 2018; Pillay et al. 2020; Stevenson et al. 2020).

In the aforementioned studies, the EEG is typically segmented into windows of 30 to 60 s, in which the EEG signal is often assumed to be stationary (e.g., for Fourier-based methods). However, the dynamics of brain activity in fact exhibit intricate non-stationary behaviour. Microstate (MS) analysis of EEG signals provides, without any a priori assumption, a comprehensive perspective on the activity of the entire cortex by identifying short periods of quasi-stable states (microstates), characterized by quasi-stable topographies (MS maps) of electric potential on the scalp (Murray et al. 2008). The typical duration of these quasi-stable states is in the order of 100 ms. The MS maps reflect the activity of distributed cortical networks, and the transition from one MS to another suggests changes in the global brain network activity (Lehmann et al. 1987). The MS analysis framework models the EEG as a sequence of microstates, which allows characterizing the global brain activity and dynamics through specific MS sequences (Khanna et al. 2015; Michel and Koenig 2018). This data-driven approach provides an informative framework for studying changes in the activity of the multiple brain networks. Moreover, unlike conventional EEG analysis techniques that assess the brain activity at specific electrode locations, during specific time intervals, and within given frequency bands, MS analysis offers a global view of the brain activity and its dynamics.

MS analysis has been used in adult populations to investigate global brain activity alterations in various neurological and psychiatric diseases, such as dementia, Alzheimer’s disease, schizophrenia, bipolar disorders, stroke and multiple sclerosis (Brown and Gartstein 2023; da Cruz et al. 2020; Nishida et al. 2013; Vellante et al. 2020; Zappasodi et al. 2017). Studies on brain maturation were conducted in paediatric and adult populations to assess normative microstate data (Koenig et al. 2002) and to investigate age and sex related changes of the temporal dynamics of EEG microstates (Tomescu et al. 2018). However, these studies only considered children from 6 years of age onwards. Indeed, the application of MS analysis in preterm neonates is limited. Khazaei et al. studied microstates in neonatal background EEG and were able to discriminate between sleep states using MS analysis (Khazaei et al. 2021). Another study used MS analysis to characterize the brain response to pain in preterm and term neonates (Rupawala et al. 2023). In the latter, event-related potentials with an epoch length of 2 s were studied.

To our knowledge, no research exists that explored the use of microstates to analyse brain maturation from neonatal background EEG. Therefore, the aim of this study was to assess whether MS analysis can evidence changes in the global dynamics of the preterm neonatal brain during the first weeks of life that can be related to maturation. To this aim, microstates were extracted from the EEG recordings of a cohort of preterm neonates with different post-menstrual ages and normal neurodevelopmental outcome, and the relation between MS features and the brain age was explored.

## Materials and methods

### Dataset

**Fig. 1:**
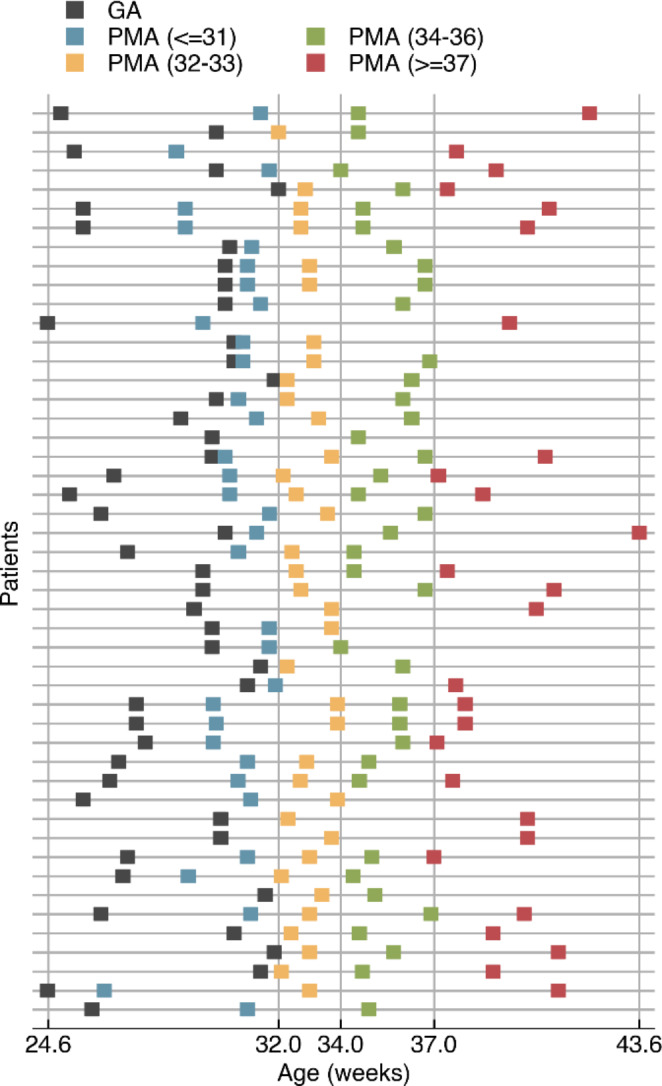
Overview of the dataset, illustrating the timings of the EEG recordings. Each row represents a neonate. The grey marker indicates the gestational age (GA, i.e. the age at birth), and the remaining markers represent the post menstrual ages (PMA) at the time of the included EEG recordings, where the colors indicate the age category to which each recording belongs

Data was recorded from the NICU of the University Hospitals, Leuven (Belgium) in accordance with the relevant guidelines and regulations and approved by the ethics committee of the University Hospitals, Leuven. All neonates were recruited after informed consent from the parents. For this retrospective data analysis, the anonymized data of 48 preterm neonates (23 females, 25 males) were included. The neonates were born between 24.6 and 32.0 weeks of gestational age (GA), see Fig. 1. All included neonates had a normal neurodevelopmental outcome at nine months of age, defined according to the Bayley Scores of Infant mental and motor Development II (BSID-II) (Dereymaeker et al. 2017a). EEG was recorded using BrainRT OSG Equipment (Mechelen, Belgium) at a sampling rate of 250 Hz using the standard 10–20 electrode system with nine channels (Fp1, Fp2, C3, C4, T3, T4, O1, O2 and Cz) (Jasper 1958). Each neonate had multiple recordings (median of 3 recordings) at various post-menstrual ages (PMA) ranging from 26.4 to 43.6 weeks. The PMA is the age of the neonate at the time of recording, counted from the first day of the mother’s last menstrual period. The timings of the recordings are visualized in Fig. 1. In total, 135 EEG recordings were included, and the median duration of a recording was 4.4 h. The recordings were categorized into age groups based on PMA at the time of recording. Based on previous studies and aiming for groups with a similar number of recordings, we defined four age groups based on PMA (De Wel et al. 2017; Lavanga et al. 2017): ≤31 weeks (n = 34), 32–33 weeks (n = 36), 34–36 weeks (n = 38), ≥37 weeks (n = 27). When assigning the EEG recordings to these groups, we made sure that no neonate had more than one recording in an age group (see Fig. 1).

### Sleep classification, preprocessing and epoch selection

MS analysis was not done on the entire raw EEG recordings, but on carefully selected 5-minute epochs. To manually select appropriate epochs from each recording, we first classified sleep states in the EEG and subsequently pre-processed the data. This is explained in more detail below.

**Fig. 2:**
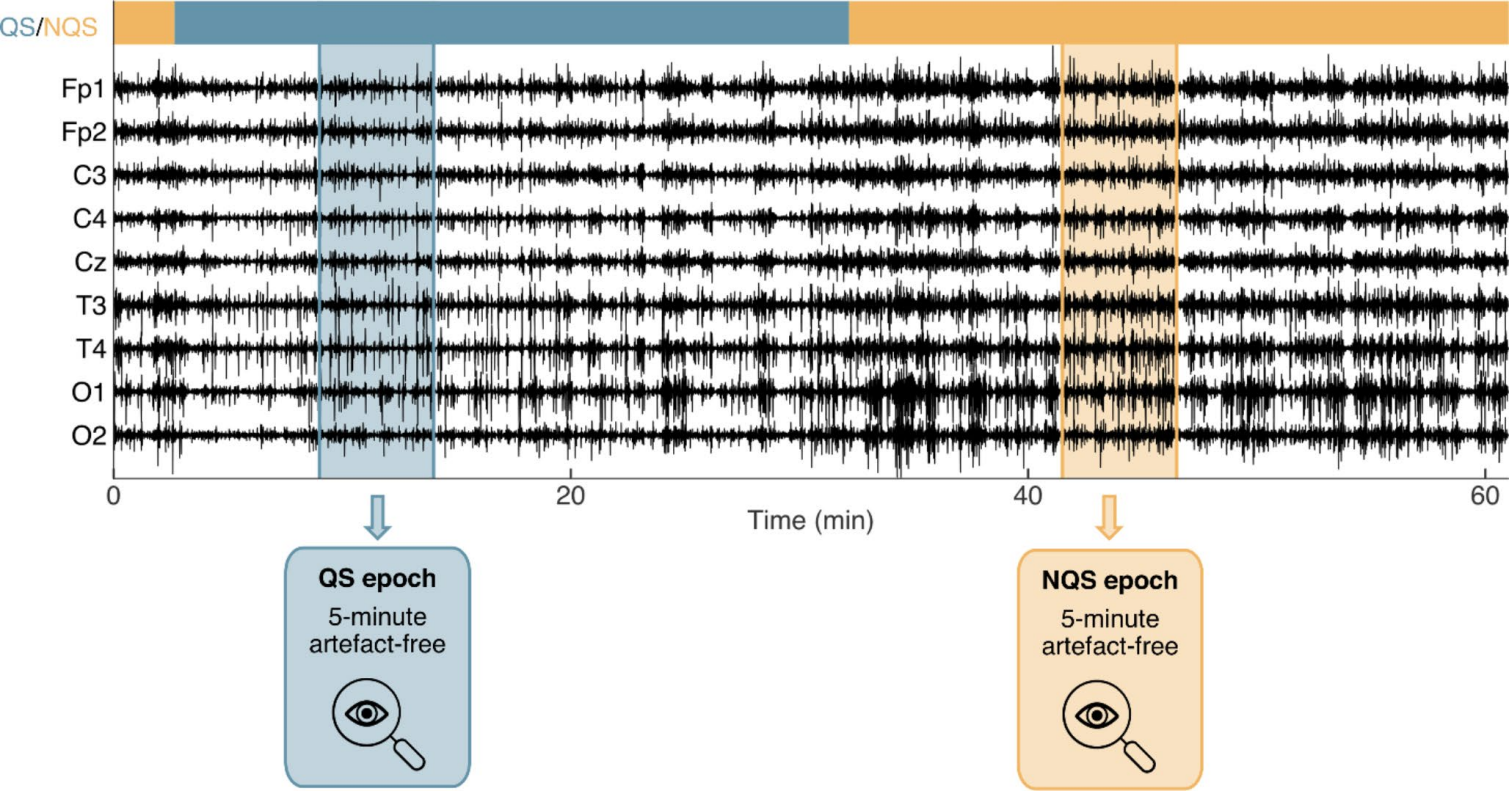
Epoch selection procedure. The sleep state was automatically detected using the method of Ansari et al. (Ansari et al. 2020), and its results are visualized by the coloured bar at the top. Here, blue indicates quiet sleep (QS) and yellow non-quiet sleep (NQS). Next, a QS and a NQS epoch of 5 min was manually selected for further analysis, verifying that they were free from artefacts (by visual inspection). Note: for visualization purposes only a part of the EEG recording is shown, of which the total length was 3.6 hours

Neonates spend most of their time asleep, and as in adults, different sleep states can be distinguished during a sleep cycle. There are two main sleep states in neonates: active sleep (AS) and quiet sleep (QS). Compared to adults, active sleep is related to rapid eye movement (REM) sleep, whereas quiet sleep is related to non-REM sleep. Among other physiological signs such as breathing rate and heart rate, brain function and EEG dynamics differ between sleep states. For this reason, differentiating sleep states when analysing neonatal EEG is important when studying brain maturation (Dereymaeker et al. 2017a). In the context of MS analysis, this was recently confirmed in a study by Khazaei et al., who found that neonatal MS features differ between AS and QS (Khazaei et al. 2021). Therefore, it is important to separately analyse EEG data obtained in different sleep states. In this study, we used an automated sleep classifier to identify parts in the data that correspond to QS (Ansari et al. 2020). All parts that were not identified as such, are referred to as non-quiet sleep (NQS). Therefore, this sleep classifier classifies each moment of the EEG recording as either QS or NQS (see Fig. 2 at the top). The NQS parts can either correspond to AS or wakefulness. The model does not distinguish AS from wakefulness, which was a deliberate choice by the authors of the sleep classifier for the reason that AS and wakefulness without movements/artefacts have similar EEG characteristic (Ansari et al. 2020). However, since neonates spent most of their time asleep, the non-quiet sleep data can be assumed to consist predominantly of AS.

After identifying the sleep states in the recording, the raw EEG data was pre-processed to facilitate MS analysis. The EEG was first bandpass filtered (7th order Butterworth) to reduce noise and artefacts. The low-pass frequency was set to 25 Hz (Rupawala et al. 2023), and the high-pass frequency was set to 0.2 Hz to preserve the delta frequency band which is dominant in neonatal EEG (0.5-4 Hz) (Finn et al. 2019; Khazaei et al. 2021; van ’t Westende et al., 2022). Second, the EEG was resampled to 100 Hz to speed up subsequent computations. Third, the EEG was re-referenced to the common average, which is common practice in MS analysis (Murray et al. 2008; Pascual-Marqui and Lehmann 1993). An example of this pre-processed EEG is shown in Fig. 2.

After preprocessing, two artefact-free epochs (each of 5 min duration) were selected from each EEG recording for subsequent MS analysis: one epoch during quiet sleep (QS), and the other one during non-quiet sleep (NQS). We selected these epochs such that they were free from movement or recording artefacts (by visual inspection). This epoch selection procedure is illustrated in Fig. 2. For the group analysis explained below, each selected EEG epoch was assigned to one of eight groups, based on the PMA and the sleep state (4 age groups x 2 sleep states), i.e., <=31-QS, <=31-NQS, 32-33-QS, 32-33-NQS, etc.

### Microstate analysis

MS analysis was performed on the extracted 5-minute epochs of the 9-channel EEG. To determine the optimal number of microstates (k), we did the clustering for varying k (ranging k from 3 to 15) and looked for the elbow-point in the dispersion (a clustering-quality metric), according to the Krzanowski-Lai (KL) criterion (Murray et al. 2008).

To represent the EEG data as a sequence of microstates, MS analysis generally requires two steps, i.e., determination of the MS maps, and (back)fitting the MS maps to the EEG data. For the MS maps we can either use individual maps (i.e., dominant topographies found within one specific epoch), or group-level maps (i.e., dominant topographies found within a specific group of epochs, e.g., all QS epochs of one specific age group). For the analysis of map-specific MS metrics (such as the average duration of one particular MS), the use of group-level (or global) maps is generally more reliable (Khanna et al. 2014). However, a group-level approach suffers from the limitation that sometimes not all recordings in the group can be adequately modelled by a common set of MS maps. This limitation can be solved by using individual maps, where the optimal set of MS maps is found for each individual EEG recording. The limitation of using individual maps is that only individual map metrics can be reliably studied, i.e., metrics that are not specific to one particular MS, such as the average duration of all microstates. Given that both approaches have their advantages and disadvantages, we included both approaches in this study. More concretely, we did a group-level analysis making use of group-level maps to investigate the relation between age group and map topographies, map-specific metrics, and transitions. Then, we also performed an individual analysis making use of individual maps to study the relation between age and individual map metrics. All computations were performed in MATLAB using custom code and the microstates plugin for EEGLAB, written by Thomas Koenig (https://www.thomaskoenig.ch/index.php/software/microstates-in-eeglab).

**Fig. 3:**
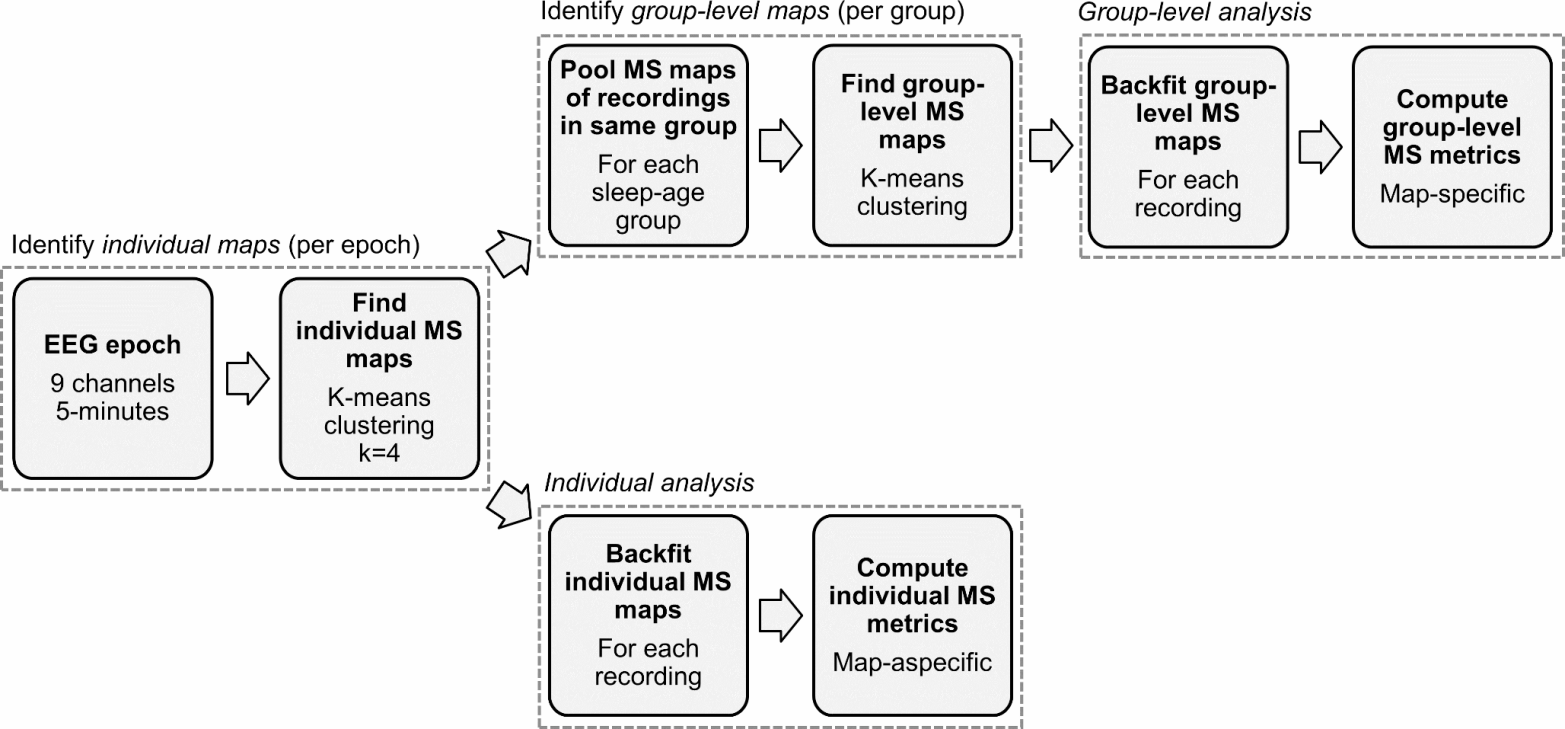
Microstate analysis pipeline that shows how a 5-minute EEG epoch is processed

The pipeline for obtaining the MS results is illustrated in Fig. 3. It consists of four blocks. In the first block, individual MS maps are identified per data epoch. To this end, a modified k-means clustering method was applied to each EEG epoch to find the dominant microstates at an individual level (Pascual-Marqui et al. 1995). The input to this clustering was a matrix with EEG data where the first dimension corresponds to different time-points in the EEG epoch and the second dimension to the nine channels. Here, only the time-points at global field power (GFP) peaks were used for clustering and polarity was ignored by the modified clustering method. This resulted in the identification of MS maps in each 5-minute EEG epoch (referred to as individual maps).

In the second block, the group-level MS maps are identified, i.e., per age-sleep group (e.g., all QS epochs from a specific age group). To obtain the group-level maps, the individual maps were pooled per age-sleep group and from the pooled MS maps the dominant group-level MS maps were identified for each group (using the same modified k-means algorithm as before). More specifically, the input to this clustering step was an EEG data matrix obtained by concatenating all individual maps of all epochs in the group. This step yielded group-level maps for each of the eight age-sleep groups. The order of the group-level maps was matched among groups by finding an ordering of the maps that maximized the average spatial correlation between corresponding maps among groups (Khazaei et al. 2021). To quantify how well these group-level MS maps were able to model the EEG data, the global explained variance (GEV) was computed per group (Murray et al. 2008).

In the third block, the group-level analysis is done. Here, the group-level maps are backfitted onto the entire continuous EEG epoch (i.e., not only on the GFP peaks), for all the epochs in the corresponding group. This backfitting procedure assigns a MS to each time point in the EEG data by selecting the MS whose topography has maximum spatial correlation with the recorded scalp potentials at that time point. Additionally, in this backfitting procedure a smoothing procedure was applied as described by Pascual-Marqui et al., using a time window of 30 ms and a non-smoothness penalty factor of 1 (Pascual-Marqui et al. 1995). The backfitting produces a sequence of microstates, from which a set of MS metrics was computed for each epoch. These MS metrics include MS duration (in seconds; it is an index of stability of the underlying brain dynamics), MS occurrence (in Hz; it expresses the tendency of the underlying neural sources to become active and dominant), and MS coverage (in %; it indicates the relative predominance of the active sources underlying the given MS) (Khazaei et al. 2021; Lehmann et al. 2005). In the group-level analysis, these metrics are computed per MS.

In the fourth block, the individual analysis was done. Here, the individual maps were backfitted to the EEG data of the corresponding epoch, with the same method as described in the previous paragraph. Likewise, a set of metrics, including MS duration and MS occurrence, was computed to characterize the MS sequence. Unlike for the group-level analysis, in this individual analysis, these metrics are not computed separately per MS, but instead individual map metrics are obtained by computing the average duration and occurrence of all microstates. For this reason, coverage was not included, since coverage is always 100% when considering all microstates. Besides duration and occurrence, we computed the Hurst exponent of the MS sequence, which is another individual map metric which captures the degree of temporal dependence (Tait and Zhang 2022a; Van De Ville et al. 2010). The Hurst exponent was estimated using detrended fluctuation analysis using the +microstate toolbox for brain MS analysis in sensor and cortical EEG/MEG (plus-microstate.github.io) (Tait and Zhang 2022b).

### Statistical Analysis

We analysed the effect of maturation on brain dynamics by analysing the obtained MS features and their relation with PMA. The MS analysis is divided into two main parts: group-level analysis and individual analysis. In the group-level analysis, we find and analyse group-level MS maps and their corresponding MS sequences. In the individual analysis, we analyse the MS sequences obtained with individual maps.

The group-level analysis consists of three sections: MS topography, MS metrics, and MS syntax. First, it was investigated whether the topography of the dominant MS varied with PMA. To this end, the similarity of dominant group-level MS maps was compared between groups by means of topographical analysis of variance (TANOVA) using the spatial correlation between the maps as effect size (Koenig and Melie-García 2009). This TANOVA is a randomized permutation test, whereby the channels of the maps are randomly permuted. We repeated this permutation 5000 times to get an empirical distribution of the random effect size, against which the actual effect size is tested for significance, at a significance level of $\alpha =0.05$.

Second, we analysed the effect of PMA on MS duration, occurrence and coverage. The dataset consisted of repeated measurements of the neonates at different PMAs. However, not all the data in each age group comes from the same patients, as this is a retrospective study and EEGs were not always obtained in all age groups. To account for such missing data within a repeated measures dataset, a mixed effect model was applied. Here, fixed effects were PMA age group, sleep state, MS and their interactions. The patient-dependence was accounted for by adding a random intercept as a random effect to the model. The results of the mixed effect model (using SPSS) are reported along with the estimated marginal means.

Patterns in the MS sequence obtained with the group-level maps (MS syntax) were analysed in two ways. First, we performed a randomized chi-square test with 5000 repetitions to test the hypothesis that the syntax is random as described by Lehmann et al. (Lehmann et al. 2005). Second, the hypothesis that the observed transition probabilities between any two MS is due to chance was tested within each group by means of a paired t-test between the observed and expected transition probabilities for each possible transition (normality assumption was fulfilled). More concretely, for each group and each possible MS transition, we tested whether the difference between the expected and observed transition probability was different from zero. The expected and observed transition probabilities are computed as in Lehmann et al. (Lehmann et al. 2005). To account for multiple testing, we corrected the p-values using the False-Discovery-Rate (FDR) controlling procedure.

Besides a group-level analysis using the group-level maps, we did an individual analysis using the individual maps. In this final analysis, we tested how age is related to individual map metrics (mean duration, mean occurrence and Hurst exponent). We used a mixed effects model to quantify the relationships. Given that in this analysis we use individual maps instead of group-level maps, we used PMA as a continuous age variable instead of the discretized age groups. In the mixed effects model, fixed effects were PMA, sleep state, and their interaction. Like in the group-level analysis, the patient-dependence was accounted for by adding a random intercept as a random effect to the model. The results of the mixed effect model (using SPSS) are reported, as well as the Pearson correlation coefficients between the individual map metrics and PMA.

## Results

### MS topography

**Fig. 4:**
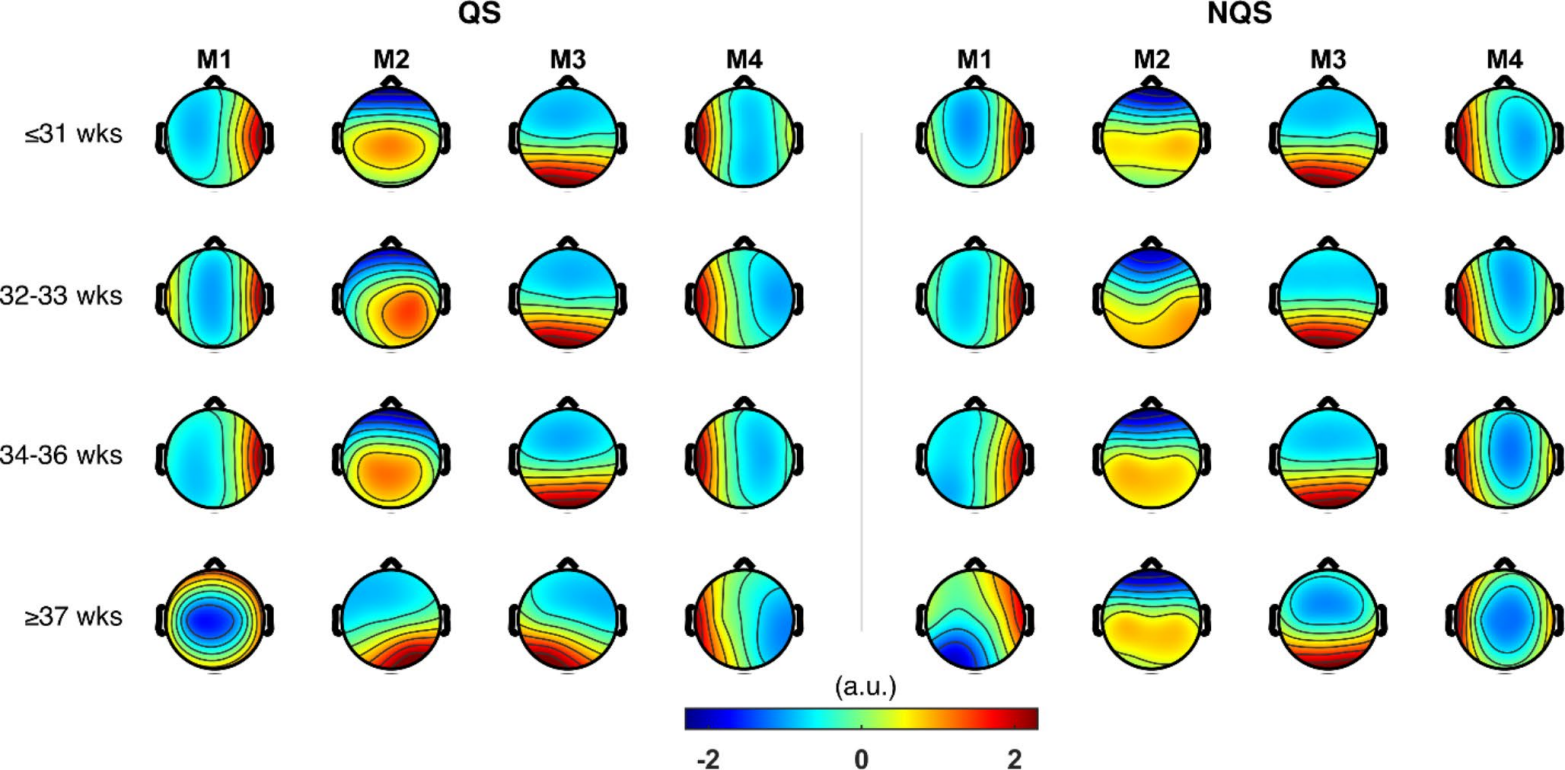
The four MS maps (M1, M2, M3, M4) for each age group and sleep state. Left: quiet sleep (QS); right: non-quiet sleep (NQS)

**Fig. 5:**
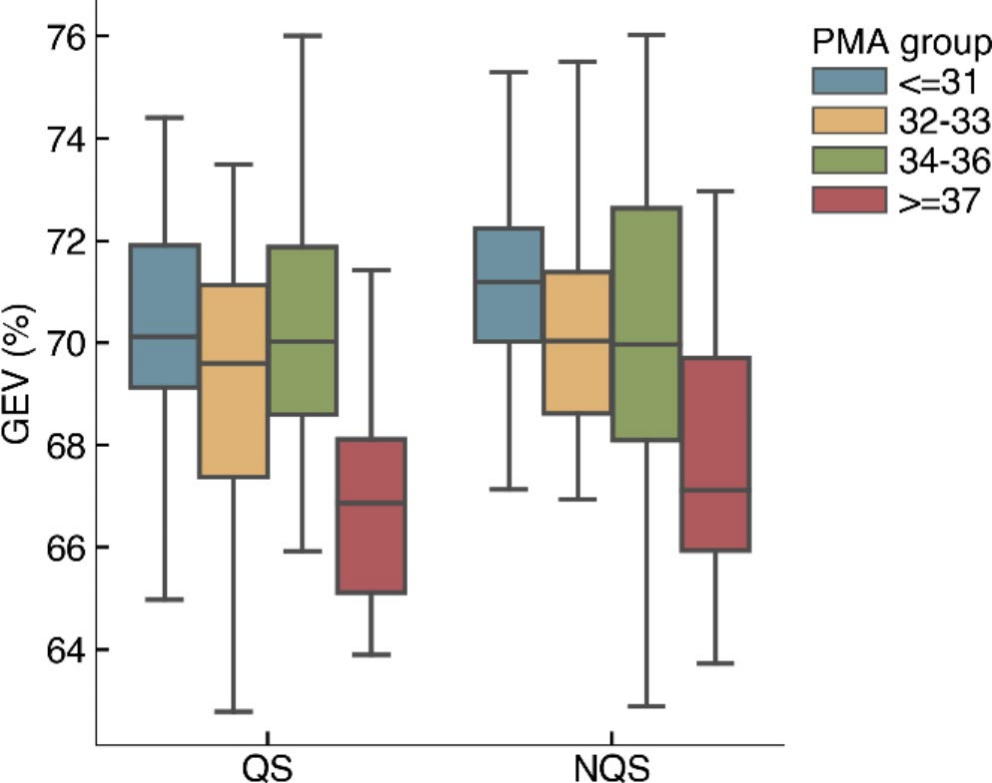
Global explained variance (GEV) per group using the group-level MS maps

According to the KL criterion, we found that four MS maps $(k=4)$ was optimal, and this was the same for all age groups and sleep states. Figure 4 shows the group-level MS maps and Fig. 5 shows the GEV for each group. Using the group-level maps, the mean GEV (± SD) across all recordings was 69.5 (± 3.0) %. The GEV drops from around 70–67% in the oldest age group.

**Fig. 6:**
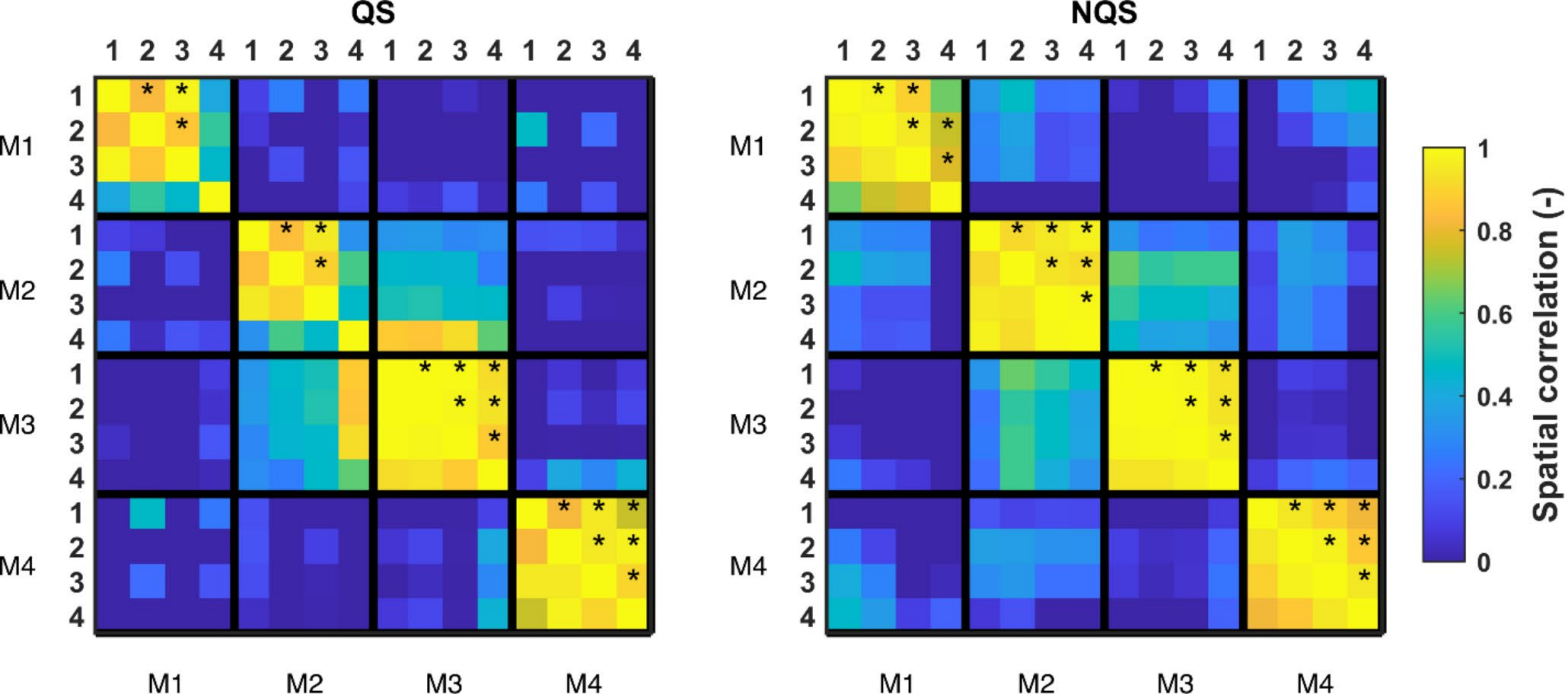
Spatial correlation between map topographies of different age groups (1: <=31 weeks, 2: 32–33 weeks, 3: 34–36 weeks, 4: >=37 weeks). High values indicate similar maps, while low values indicate dissimilar maps. *p < 0.05 (TANOVA permutation test), i.e., the maps significantly correlate (spatially). For clarity, significance is only tested between the same microstates (i.e., only TANOVA tests were carried to compare a specific MS (MS1, MS2, MS3, or MS4) between age groups). Because the matrix is symmetric, significance is only indicated in entries above the diagonal

Visually comparing the maps between age groups in Fig. 4 concludes that the maps remain similar between age groups. Figure 6 confirms this as it compares the topographies of each microstate among the four age groups (1, 2, 3 4). Comparisons annotated with an asterisk indicate that those MS maps are significantly similar (i.e., spatially correlated). Only the oldest age group (PMA > = 37 weeks) has microstates maps that significantly differ from the other three age groups: for QS, maps M1 and M2 are different from maps M1 and M2 in the other age groups, and for NQS map M1 is different from M1 in the other age groups.

### MS metrics

**Fig. 7:**
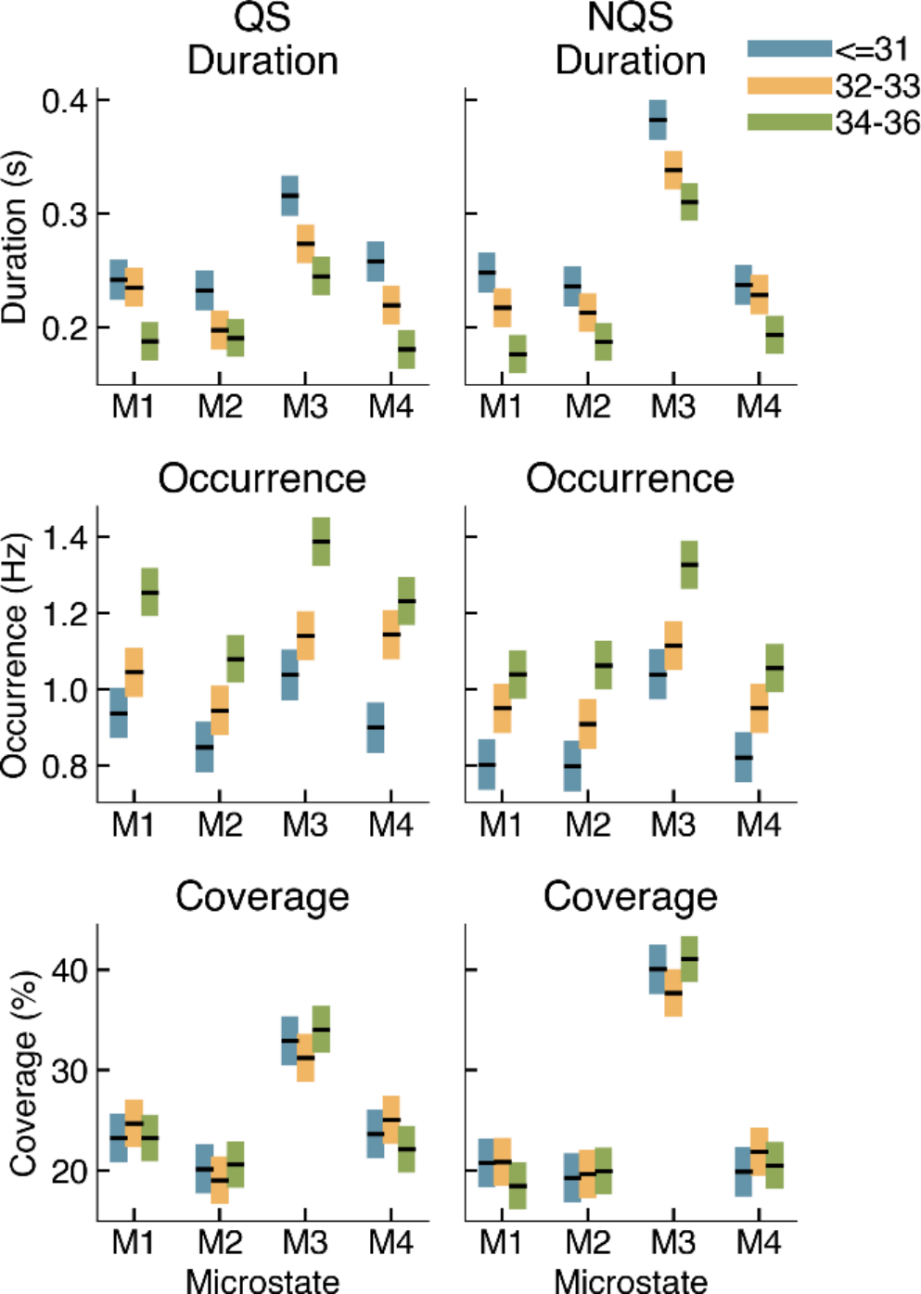
Estimated marginal means for mean microstate duration, occurrence and coverage for quiet sleep (QS) and non-quiet sleep (NQS). The boxes represent the upper and lower (95%) confidence limit for the marginal means. Here, the oldest age group is excluded because its MS maps differ from the MS maps of the other groups

A mixed effects model was applied to check for differences in MS metrics between age groups. We excluded the oldest age group from this mixed effects model, because its MS maps differed from the other age groups. Table 1 shows the statistical summary of these mixed effects models and Fig. 7 shows the marginal means of the mixed effects models for duration, occurrence and coverage for each age group, sleep state and MS map. These results show that age group and sleep state have significant effects on the MS metrics (Table 1). For example, in Fig. 7, a clear trend with PMA is seen for duration and occurrence. MS duration decreases and MS occurrence increases with PMA, and this relationship is similar across microstates and sleep states, as evident from Fig. 7. Trends for coverage are less obvious in Fig. 7. However, the mixed effects model summary in Table 1 indicates a significant interaction term for age group and microstate.

**Table 1:** Results of the mixed effect models for MS metrics (duration, occurrence and coverage). Here, the oldest age group is excluded because its MS maps differ from the MS maps of the other groups

	*df*	*F*	*p*
*Duration*			
Age group	2, 827.49	118.83	< 0.001
Sleep state	1, 794.10	27.10	< 0.001
Microstate	3, 794.10	244.94	< 0.001
Age group x Microstate	6, 794.10	2.26	0.036
Sleep state x Microstate	3, 794.10	29.97	< 0.001
Age group x Sleep state	2, 794.10	0.12	0.887
Interaction^1^	6, 794.10	1.65	0.132
*Occurrence*			
Age group	2, 835.16	180.27	< 0.001
Sleep state	1, 794.21	60.89	< 0.001
Microstate	3, 794.21	73.97	< 0.001
Age group x Microstate	6, 794.21	2.41	0.026
Sleep state x Microstate	3, 794.21	8.61	< 0.001
Age group x Sleep state	2, 794.21	1.68	0.187
Interaction^1^	6, 794.21	1.28	0.263
*Coverage*			
Age group	NA	NA	NA
Sleep state	NA	NA	NA
Microstate	3, 840	276.14	< 0.001
Age group x Microstate	6, 840	2.71	0.013
Sleep state x Microstate	3, 840	28.06	< 0.001
Age group x Sleep state	NA	NA	NA
Interaction^1^	6, 840	0.451	0.845

NA: computation for group effect is not applicable since the total coverage is 100%

^1^Interaction of all three terms: Age group x Sleep state x Microstate

### MS syntax

**Fig. 8:**
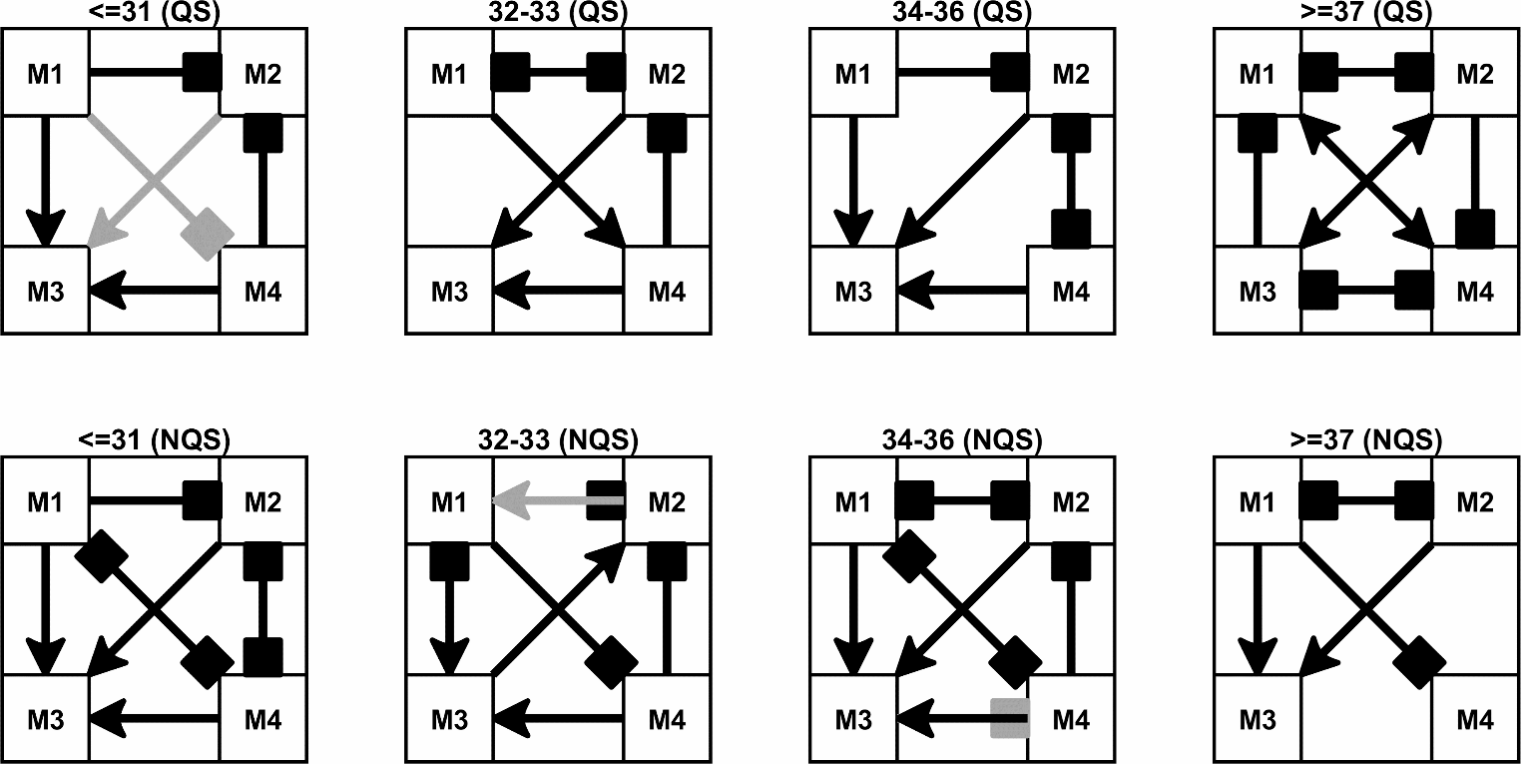
Significant transitions between microstates for each age group and sleep state. Top: quiet sleep (QS), bottom: non-quiet sleep (NQS). Arrow heads indicate more observed transitions than expected and square arrow heads indicate less observed transitions than expected. Grey and black arrows indicate p < 0.1 and p < 0.05, respectively

To test whether the sequence of microstates – the MS syntax – is random or not, a chi-square test was performed, and its results are presented in Table 2. The p-values in this table are all less or equal to 0.0006, indicating that the MS syntax is not random and that there is a structure in the sequence of microstates. Figure 8 visualizes the transitions among the microstates. In this figure, the transitions that occur significantly less or more frequently than expected at random are drawn. The transition patterns remain fairly stable across age groups, especially for the three youngest groups. For instance, frequent transitions into M3 from several microstates is a recurring pattern in both QS and NQS. An opposite recurring effect regards M2, for which we observe less frequent transitions. The largest change in transition patterns is seen when comparing the two oldest age groups. Here, some of the patterns that were stable for the three youngest groups change, such as the transitions between M4->M2 and M4->M3 during both QS and NQS.

**Table 2:** P-values for randomized chi-square test to test if the MS syntax is random for each sleep state and age group

	*<=31*	*32–33*	*34–36*	*>=37*
*QS*	p = 0.0006	p < 0.0002	p < 0.0002	p < 0.0002
*NQS*	p < 0.0002	p < 0.0002	p < 0.0002	p < 0.0002

### Individual map metrics and PMA

**Fig. 9:**
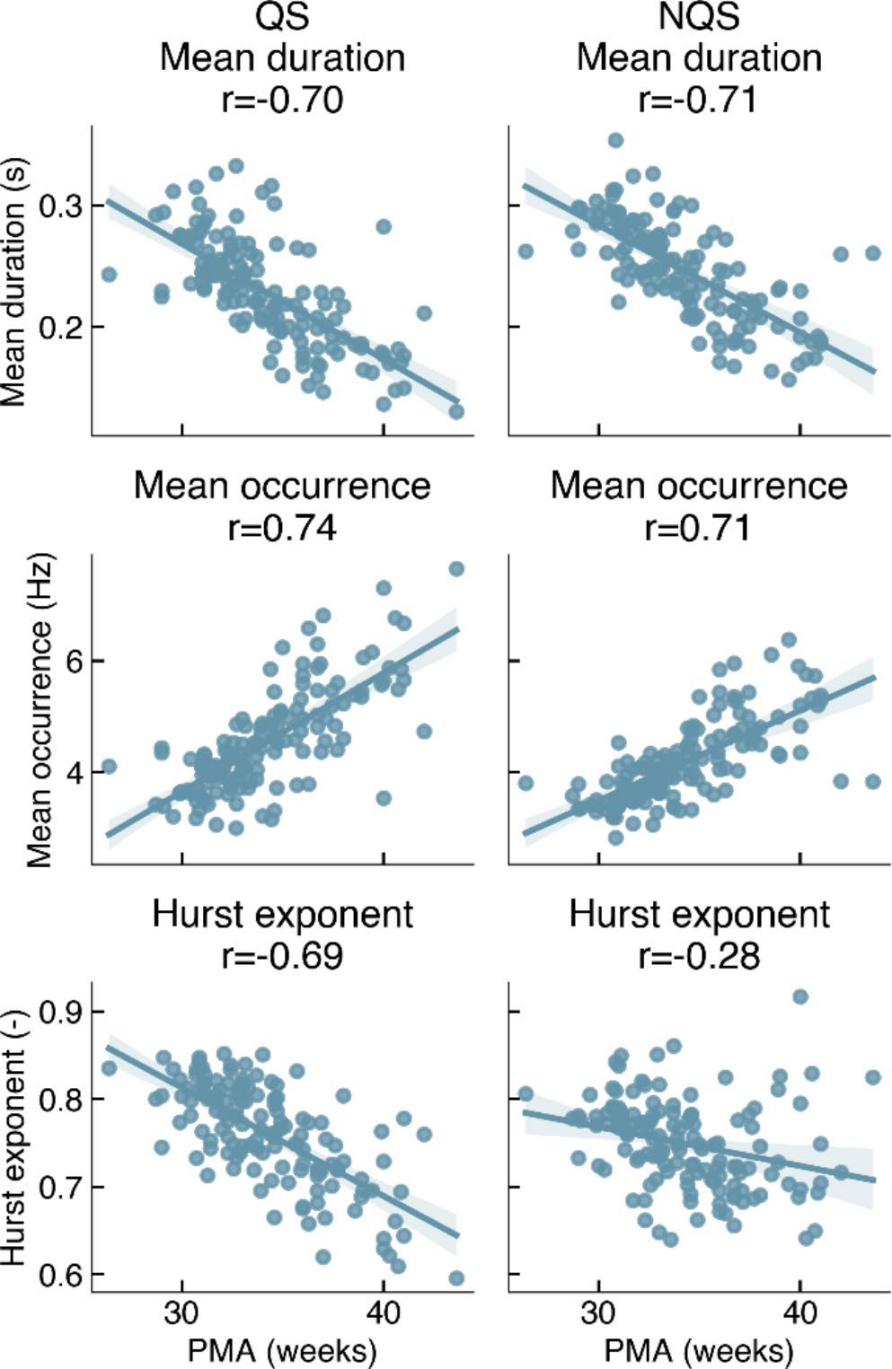
Relationships between individual map metrics and PMA. r: Pearson correlation coefficient

Due to differences in group-level maps, the oldest age group was excluded from the group-level analysis of the MS metrics (Table 1). To evidence effects of age on microstate metrics including also the group of the oldest neonates, we applied a mixed effects model to individual map metrics obtained from individual MS maps (mean duration, mean occurrence and Hurst exponent). The results are presented in Table 3. For duration, we found a significant effect of PMA [F(1, 249.907) = 293.846, p < 0.001], but not significant effect of sleep state (p > 0.1) or PMA x sleep state interaction (p > 0.1). The negative coefficient (-0.010) indicates that the mean duration significantly decreased with age. This is also visible in Fig. 9, which shows how the average duration of a microstate decreases with PMA, with a correlation of -0.70 and − 0.71 for QS and NQS, respectively.

For occurrence, the most significant effect was age [F(1, 252.949) = 315.645, p < 0.001]. Here, age x sleep state interaction was also significant [F(1, 220.619) = 6.159, p = 0.014], and sleep state was almost significant [F(1, 220.619) = 3.748, p = 0.054]. Mean occurrence significantly increased with age, and occurrence in QS was higher than in NQS (Fig. 9). Moreover, the correlation values of occurrence vs. PMA were 0.74 and 0.71 for QS and NQS, respectively.

The Hurst exponent was significantly related with age, sleep state and their interaction (p < 0.001), see Table 3. Figure 9 shows that the Hurst exponent was > 0.5, indicating long-term correlations in the MS sequence. With age, these long-term correlations decreased significantly, especially in QS (r=-0.69). For NQS, the Hurst exponent reduced to a lesser extent and was less strongly correlated with age, though still significantly (r=-0.28, p < 0.001).

**Table 3:** Results of the mixed effect models for individual map metrics (mean duration, mean occurrence and Hurst exponent), obtained with individual MS maps. Values for Lower and Upper 95% define the 95% confidence interval

	*df*	*F*	*p*	*Coefficient*	*Lower 95%*	*Upper 95%*
*Mean duration (s)*						
Age (PMA)	1, 249.907	293.846	< 0.001	-0.010	-0.011	-0.008
Sleep state (NQS)	1, 220.173	0.021	0.884	-0.005	-0.076	0.066
Age*Sleep state	1, 220.173	0.428	0.514	0.001	-0.001	0.003
*Mean occurrence (Hz)*						
Age (PMA)	1, 252.949	315.645	< 0.001	0.215	0.185	0.244
Sleep state (NQS)	1, 220.619	3.748	0.054	1.379	-0.025	2.782
Age*Sleep state	1, 220.619	6.159	0.014	-0.051	-0.092	-0.011
*Hurst exponent (-)*						
Age (PMA)	1, 259.942	97.359	< 0.001	-0.012	-0.015	-0.010
Sleep state (NQS)	1, 222.410	23.846	< 0.001	-0.282	-0.396	-0.168
Age*Sleep state	1, 222.410	22.258	< 0.001	0.008	0.005	0.011

We further checked whether adding GA or sex were confounding factors. Correlations between GA and the individual map metrics were not significant. Furthermore, we did not find any significant differences for male vs. female. Additionally, a multiple linear regression analysis performed on the QS data with PMA, GA and sex as independent variables showed that only the PMA coefficient was significant for predicting mean duration, occurrence and Hurst exponent.

## Discussion

We explored the use of MS analysis to study the functional brain development (otherwise called brain maturation) in preterm neonates. We found that the dynamics of the brain activity in preterm neonates, as recorded in the EEG, can be modelled with similar MS topographies in both QS and NQS from the 31st to the 36th week PMA. Changes in the MS topography occurred after 37 weeks PMA, as well as changes in the pattern of transitions between microstates. These changes might reflect functional reorganization or structural/functional development of novel cortical networks when the preterm neonate approaches the PMA typical of term birth. This observation aligns with trends in brain development that have been described in the literature, which suggest that the second half of gestation (i.e., 20 to 40 weeks) and the early postnatal period are critical for the formation of functional neural networks (Kinney and Volpe 2012; Kostović and Jovanov-Milošević 2006; Tau and Peterson 2010).

The changes observed in the MS topography after 37 weeks PMA are reflected in the GEV. In fact, using four (group-level) MS maps, we could describe approximately 70% of the preterm EEG data in our dataset. This GEV is similar to the reported GEV in adult studies and in a previous study with term neonates (Khazaei et al. 2021). When looking at the difference of GEV between the groups, we noticed that the GEV remained around 70% for the three youngest age groups. In the oldest age group, the GEV dropped to around 67%, which indicates that the EEG in the older age group is less-well represented by four MS maps, although four maps were preferred according to the KL-criterion. It is interesting to observe that the PMA of our older age group is close to the PMA of the full-term neonates investigated by Khazaei et al. (Khazaei et al. 2021), who found that the optimal number of microstates was 7 and that the associated GEV was about 70% in both QS and NQS groups. Therefore, assessing the dependence of the optimal number of microstates able to properly describe the brain dynamics in preterm and term neonates on PMA could be an interesting topic to focus on in future studies. Moreover, additional approaches besides the KL-criterion could be explored to determine the optimal number of microstates. For example, Custo et al. 2017 combined eleven different criteria into a meta-criterion to increase the confidence in the selected number of clusters. This could prevent any bias in the analysis resulting from using too few maps to model all relevant dynamics, which may reduce the discrepancy between groups maps.

Consistently with our observation that the different MS topographies observed for the group of oldest neonates might be related to functional reorganization or structural/functional development of the neonatal brain, our results show that MS duration and occurrence are related to PMA, where duration decreases, and occurrence increases with age. Even when extracting microstates at individual level and obtaining recording-specific MS maps, the mean overall MS duration and occurrence have the same strong relationship with PMA as described before. This implies that the duration of quasi-stable electrical potentials shortens with age, regardless of the topography associated with that electrical potential distribution. This might indicate the progress of discontinuous brain electrical activity into more complex, faster changing continuous EEG as the brain matures (Pavlidis et al. 2017). The average MS duration of both QS and NQS states in our cohort of preterm neonates decreases from about 250–300 ms at 30 weeks PMA to 150–200 ms after 37 weeks PMA. Khazaei et al. (Khazaei et al. 2021) reported that the MS duration of full-term neonates was about 110–150 ms. On one hand these values are shorter than the average MS duration that we found for the older preterm neonates, and on the other hand they are much longer than average MS duration found in adults during different sleep states (40–100 ms) (Brodbeck et al. 2012), or in children of 6–7 years (about 94 ms) and adults (about 80 ms) in awake state (Koenig et al. 2002; Tomescu et al. 2018). Taken together, these data confirm the tendency of MS duration to decrease during brain maturation in a continuum from early stages to term-birth, childhood and adolescence. Our results, confirmed at individual level, may serve as a reference for future research, providing normal values for MS metrics for a range of different PMA groups, opening the way to the assessment of MS duration as a reliable biomarker of brain maturation.

Furthermore, we observed that the Hurst exponent of the MS sequence decreases with age, which indicates a decrease in the degree of persistence or long-term memory of the signal. Therefore, the EEG exhibits weaker long-term correlations as the brain maturates. This effect is stronger in QS compared to NQS (Fig. 9). It is known that the EEG patterns during QS change significantly with neonatal age. More specifically, the EEG during QS evolves from a discontinuous burst-suppression pattern (*tracé alternant*) to a more continuous pattern (*high voltage slow-waves*) as the brain matures (Dereymaeker et al. 2017b). The decreasing Hurst exponent may reflect this typical evolution of the EEG signal during QS, as the discontinuous burst-suppression pattern is likely to exhibit more long-term correlations than the continuous pattern. On the other hand, the EEG patterns during NQS change less compared to QS, which could explain why the Hurst exponent is affected to a lesser extent.

Another important result of our study is that the MS syntax is not random. This means that the co-activation of specific brain regions in the preterm brain, resulting in a specific topographic map, facilitates the subsequent co-activation of another group of brain regions in a sequential way that is compatible with time-parcelled MS sequences and that can be described by MS transition probabilities. We demonstrated that this process was specific of different ages, with most relevant changes of the transition probability patterns for the oldest age group ( > = 37 weeks). Similarly to the changes in the MS topography occurring after 37 weeks PMA, the differences observed for the transition probability patterns of the older PMA groups could be explained in terms of the functional reorganization of the MS dynamics during these last weeks of brain maturation before the neonate reaches the PMA typical of term birth. In a prior study on full-term neonates, Khazaei et al. also found non-casual directional transition sequences and preferred transitional loops in the MS sequences describing the dynamics of the full-term neonatal brain (Khazaei et al. 2021).

Like us, Khazaei et al. also studied microstates in relation to different sleep states (Khazaei et al. 2021) and, despite the different study population (term babies in the study by Khazaei et al. vs. preterm babies in our study), we both obtained different MS metrics for QS and NQS states. Although these differences between QS and NQS do not seem to be related to age (see Figure A3 in the Appendix), this finding can support the notion that a different functional organization underpins different sleep states. Khazaei et al. reported longer mean MS duration and reduced MS occurrence during QS when compared to active sleep (AS). In adults, longer MS duration was observed in non-REM sleep (QS) compared to awake (NQS) (Brodbeck et al. 2012). However, our results suggest that the differences between QS and NQS are much smaller and tend to be in the opposite direction (shorter MS duration in QS compared to NQS). Khazaei et al. related a longer MS duration in QS to a higher delta power (0.5–4 Hz) in QS compared to AS, since they observed a dependence between MS duration and occurrence and the frequency content. We also observed higher delta power in QS compared to NQS (see Figure A2 in the Appendix), but we noticed an opposite effect when studying the relative amount of the power below 0.5 Hz. This finding might mean that, in our data, the slowest waves (< 0.5 Hz) were more dominant in NQS than in QS, which could explain why MS duration tends to be longer in NQS (see Figures A1 and A2 in the Appendix). Whereas we used broadband EEG, future work could study the age effect in MS analysis performed in narrow-band EEG to potentially identify frequency bands that are less or even more sensitive to age effects. It is also worth noting that an important difference between our study and previous studies is that the sleep states were identified automatically. Especially the NQS epochs identified by the automated sleep stage algorithm (a QS identifier) may differ from the AS epochs selected by clinical experts in the study by Khazaei et al. Another important difference with this study that may explain our divergent results is that our dataset consisted of 9-channel EEG, whereas Khazaei et al. worked with 19-channel EEG.

Several studies have reported on the relationship between EEG dynamics and PMA in preterm infants. For instance, Dereymaeker et al. found a negative correlation between suppression characteristics of preterm EEG and PMA, indicating that as the brain matures, the EEG transits from a burst-suppression pattern to a more continuous pattern (Dereymaeker et al. 2016). In the context of functional connectivity, Lavanga et al. observed a negative correlation between PMA and imaginary coherence indices, which measure lagged interactions between channels (Lavanga et al. 2018). Furthermore, De Wel et al. found that the complexity of the EEG signal increases with age and reported significant correlations between complexity indices and PMA (De Wel et al. 2017). Notably, the latter two studies reported correlation values with similar magnitudes to those observed in our analysis of MS metrics and PMA. All these different approaches to analysing the neonatal EEG expose different views on brain development that converge to the idea that neonatal brain maturation implies an increasing complexity in the brain functional dynamics. Our results are consistent with these findings and demonstrate that MS analysis is a valuable tool to describe brain maturation from another perspective. Given that all these methods were used to analyse EEG recordings of preterm babies with normal developmental outcome, it would be very interesting to verify whether the converging views of these approaches on brain maturation would eventually diverge in case the EEG of preterm neonates with abnormal developmental outcome would be analysed, and whether MS analysis could be proposed as a biomarker of adverse brain maturation and long-term neurodevelopmental outcome. All these different approaches can ultimately be combined to reach a comprehensive view on the activity and dynamics of the neonatal brain. Additionally, combining features from several approaches could be used to develop accurate models to monitor brain development, such as the brain age prediction models that use a collection of features from different domains to build an accurate PMA estimator (Pillay et al. 2020; Stevenson et al. 2020). Given that each MS map is the result of the co-activation of distinct brain areas and that brain functions can be described by MS metrics and syntax, an interesting question regards the relationship between functional connectivity features or complexity indices and EEG microstates during maturation in preterm infants. Future studies should address this point and investigate whether a relationship exists between specific MS features and patterns of functional connectivity or brain complexity.

Microstates have been extensively studied in adults, and the MS maps typically found in adults have been associated with specific brain functions, such as vision, attention and cognitive control (Michel and Koenig 2018). In neonates, however, we cannot assume that any of these associations applies because the neonatal brain is still under development and its dynamics is different from that of the adult brain. It is worth noting that the order with which microstates are presented in our study is arbitrary and that the four microstates that we identified do not resemble the four microstates typically found in adults. Therefore, future research could also focus on how to interpret these microstates in terms of brain functions.

Some limitations need to be considered when interpreting our results. First, the categorisation into age groups is arbitrary: given that we aimed at having approximately equal group sizes, we depended on the availability of data, which led to the age groups spanning different age ranges. Furthermore, the fact that the microstate maps differed between age groups prevented us from using global maps that could be used across all ages: this fact – likely due to the phenomenon that we wanted to observe, i.e., brain maturation – complicated the analysis and the interpretation of the results, and led to the exclusion of the oldest age group in the mixed model analysis (Table 1; Fig. 7). To accommodate for this limitation, we performed an individual analysis, where we did not group by age and analysed individual map metrics obtained with individual maps. Studying individual map metrics obtained with individual maps allows for variations in MS topographies between recordings. Moreover, since we did not use group-level maps, we could drop the arbitrary age-categorisation and study the relationship between MS metrics and age as a continuous variable, also revealing significant relationships. In clinical applications, where the assessment of brain maturation through MS metrics might be helpful, an approach using individual maps and not categorising into age groups might be preferred, as this allows for variations in MS topographies between recordings, solving the limitation of group-level analysis which requires that the EEG is modelled by the same dominant MS maps for all groups. An alternative future direction could focus on identifying a set of global MS maps that can be used to model all data across all ages, requiring a different, non-traditional approach to find these global maps. For example, these global maps could be found by pooling all recordings from all ages and finding the dominant topographies in the pooled data set. In such an approach, the optimal number of maps could be chosen such that all (or most) recordings have an acceptable GEV. We already showed that the dominant maps change with age, therefore, it is expected that more than 4 maps will be needed to model all pooled data adequately. In other words, we expect that different subsets of the global MS maps may be dominant for different ages. In such an approach, the change of dominant topographies with age could be studied by looking at the evolution of the map coverages with age.

A second limitation of our study regards the manual epoch selection. Given that EEG data obtained from long EEG recordings in the NICU are inevitably contaminated with artefacts, we opted for manually selecting artefact-free epochs. This approach could be improved by employing automated methods for artefact detection (Hermans et al. 2023; Tamburro et al. 2022; Webb et al. 2021). Therefore, future work could focus on using existing methods for automated artefact detection and thereby automating the entire analysis pipeline. Furthermore, adding automated artefact detection to the pipeline would facilitate the investigation of the consistency of MS analysis, because all artefact-free epochs within one long EEG recording could automatically be identified, enabling studying the consistency of MS analysis.

To conclude, our results showed that the spatio-temporal dynamics of the EEG in preterm neonates can be modelled as a non-casual sequence of few microstates and that the MS metrics can detect changes in the EEG patterns due to brain maturation, indicating that MS analysis can capture and characterize the changes occurring in the developing neonatal brain. Therefore, MS analysis can be proposed as a feasible tool for studying continuous background EEG in preterm neonates to assess their brain maturation. To our knowledge, this is the first study using MS analysis to this purpose: for this reason, our results could serve as a normal reference for future research.

### Electronic Supplementary Material

Below is the link to the electronic supplementary material.

Supplementary Material 1
