# Supplementary material for: Microstate Analysis Reflects Maturation of the Preterm Brain
Source: Brain Topogr. 2023 Oct 12;37(3):461–74. doi: 10.1007/s10548-023-01008-0 (PMC11026208; doi:10.1007/s10548-023-01008-0)
Supplement: Supplementary file 1 — Supplementary Material 1 [file 10548_2023_1008_MOESM1_ESM.docx]

Appendix

Figure A1 shows an example of a QS and NQS epoch from a recording in the dataset. The recording was obtained at an age of 43.6 weeks and shows an example of strong slow waves (<0.5 Hz) during NQS.


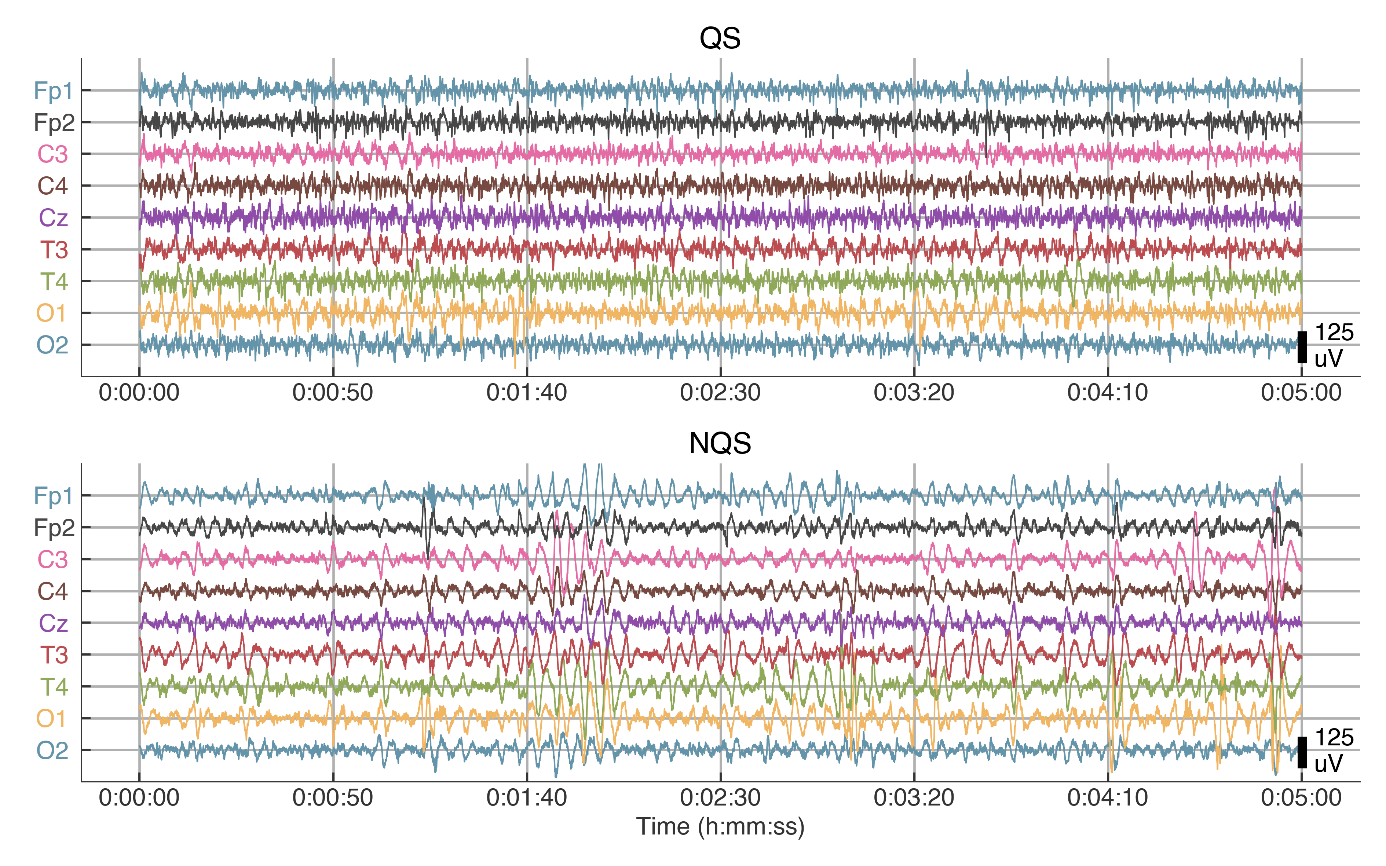


*Figure A1 Example epochs. One QS (top) and NQS (bottom) epoch from the same recording. The preprocessed EEG is shown, referenced to the common average.*

To relate the MS metrics to spectral content of the EEG, we computed relative spectral power in 5 frequency bands and the spectral edge frequency (SEF^­­­^_90_) (i.e. the frequency below which 90% of the total signal power lies). The five frequency bands are: delta_low_ (<0.5 Hz), delta (0.5 – 4 Hz), theta (4 – 8 Hz), alpha (8 – 13 Hz) and beta (13 – 25 Hz). Figure A2 shows how the MS metrics and the spectral features differ between age groups and sleep states. In Figure A3, we have shown the difference of MS metrics between sleep stages (QS-NQS) for each age group.


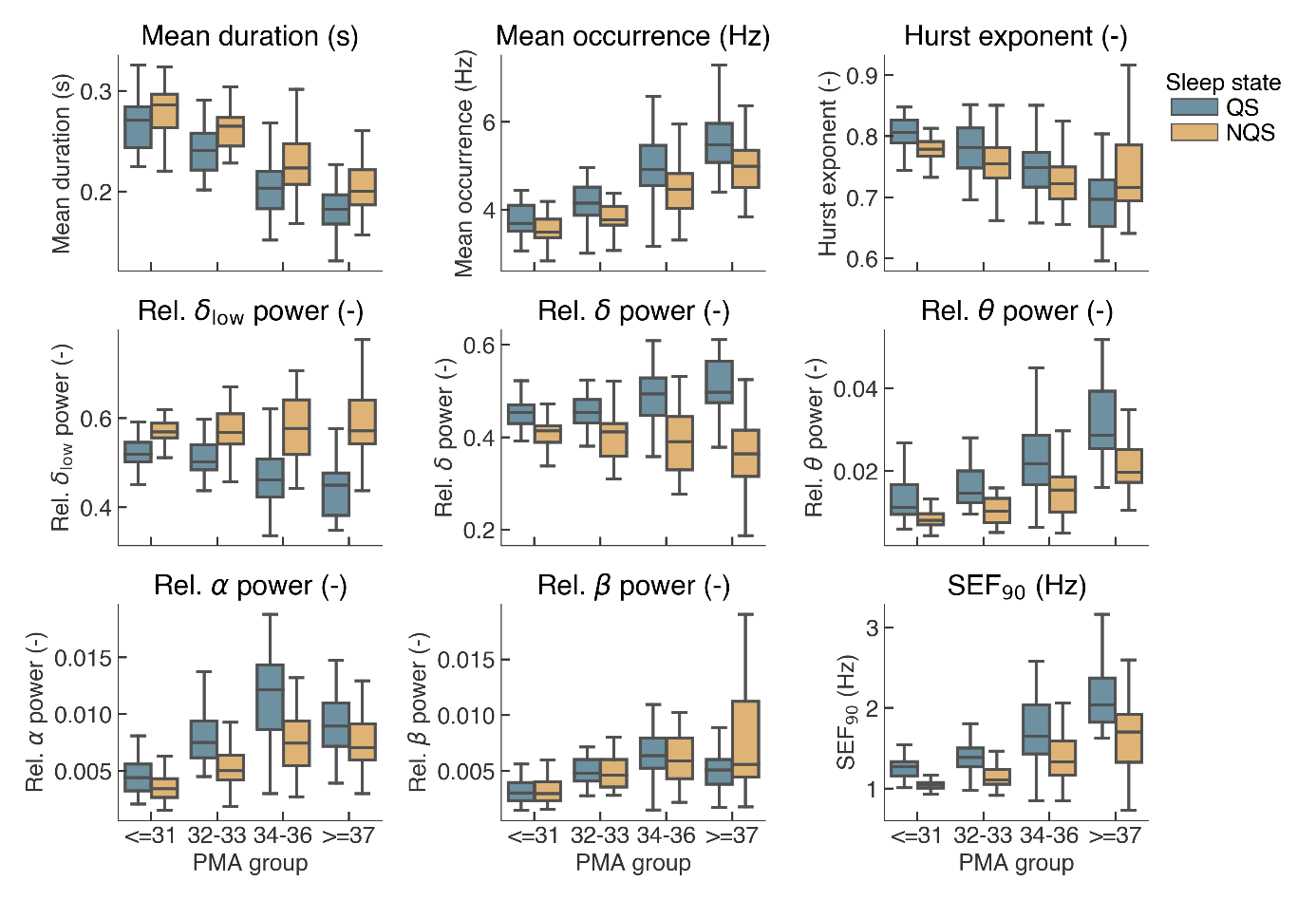


*Figure A2 Individual map metrics and spectral power features for the different age groups and sleep states.*


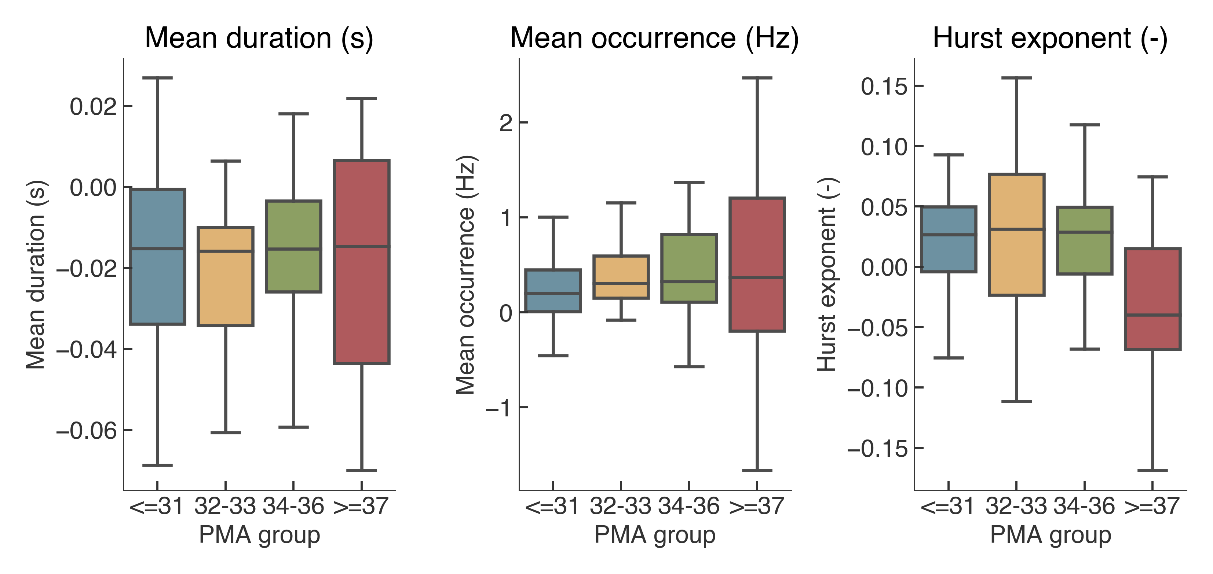


*Figure A3 The difference in individual map metrics between QS and NQS (QS-NQS), per PMA group.*
